# Early rapid local impedance drop is associated with acute lesion efficacy during pulmonary vein isolation

**DOI:** 10.1093/europace/euae260

**Published:** 2024-10-07

**Authors:** Péter Perge, Nikola Petrovic, Zoltán Salló, Katalin Piros, Vivien Klaudia Nagy, Pál Ábrahám, István Osztheimer, Béla Merkely, László Gellér, Nándor Szegedi

**Affiliations:** Heart and Vascular Center, Semmelweis University, Városmajor u. 68, 1122 Budapest, Hungary; Boston Scientific International B.V., Kerkrade, The Netherlands; Faculty of Mechanical Engineering, University of Belgrade, Belgrade, Serbia; Heart and Vascular Center, Semmelweis University, Városmajor u. 68, 1122 Budapest, Hungary; Heart and Vascular Center, Semmelweis University, Városmajor u. 68, 1122 Budapest, Hungary; Heart and Vascular Center, Semmelweis University, Városmajor u. 68, 1122 Budapest, Hungary; Heart and Vascular Center, Semmelweis University, Városmajor u. 68, 1122 Budapest, Hungary; Heart and Vascular Center, Semmelweis University, Városmajor u. 68, 1122 Budapest, Hungary; Heart and Vascular Center, Semmelweis University, Városmajor u. 68, 1122 Budapest, Hungary; Heart and Vascular Center, Semmelweis University, Városmajor u. 68, 1122 Budapest, Hungary; Heart and Vascular Center, Semmelweis University, Városmajor u. 68, 1122 Budapest, Hungary

**Keywords:** Local impedance, Atrial fibrillation, Catheter ablation, Pulmonary vein isolation

## Abstract

**Aims:**

The predictive role of local impedance (LI) drop in lesion formation using a novel contact force sensing ablation catheter was recently described. The purpose of our current study was to assess the temporal characteristics of LI drop during ablation and its correlation with acute lesion efficacy.

**Methods and results:**

Point-by-point pulmonary vein isolation was performed. The efficacy of applications was determined by pacing along the circular ablation line and assessing loss of capture. Local impedance, contact force, and catheter position data with high resolution were analysed and compared in successful and unsuccessful applications. Five hundred and fifty-nine successful and 84 unsuccessful applications were analysed. The successful applications showed higher baseline LI (*P* < 0.001) and larger LI drop during ablation (*P* < 0.001, for all). In case of unsuccessful applications, after a moderate but significant drop from baseline to the 2 s time point (153 vs. 145 Ω, *P* < 0.001), LI did not change further (*P* = 0.99). Contradictorily, in case of successful applications, the LI significantly decreased further (baseline–2 s–10 s: 161–150–141 Ω, *P* < 0.001 for all). The optimal cut-point for the LI drop indicating unsuccessful application was <9 Ω at the 4-s time point [AUC = 0.73 (0.67–0.76), *P* < 0.001]. Failing to reach this cut-point predicted unsuccessful applications [OR 3.82 (2.34–6.25); *P* < 0.001].

**Conclusion:**

A rapid and enduring drop of the LI may predict effective lesion formation, while slightly changing or unchanged LI is associated with unsuccessful applications. A moderate LI drop during the first 4 s of radiofrequency application predicts ineffective radiofrequency delivery.

## Graphical Abstract

**Graphical Abstract:**
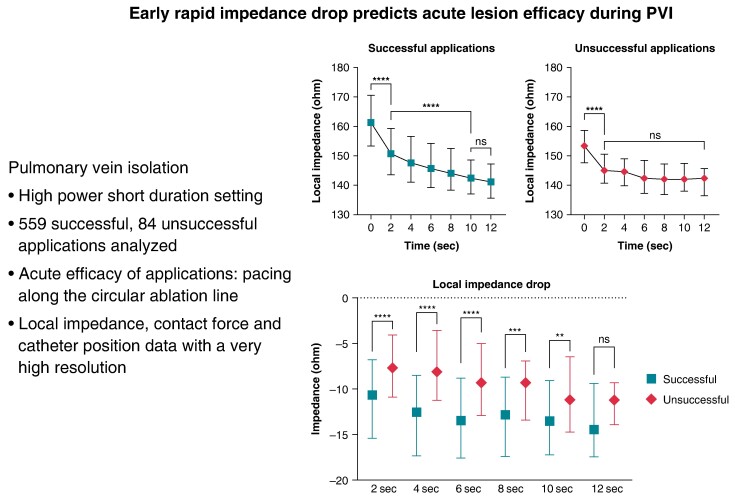

**What’s new?**

- The predictive role of local impedance (LI) drop in lesion formation using a novel contact force sensing ablation catheter for pulmonary vein isolation was recently described; however, the temporal characteristics are not established.
- The successful applications were characterized by a higher baseline LI and a larger LI drop.
- In case of unsuccessful applications, after a moderate but significant drop from baseline to the 2-s time point, LI did not change further over time. On the contrary, in case of successful applications, the LI significantly decreased further continuously
- Failing to reach the LI drop of >9 Ω after 4 s of ablation predicted unsuccessful applications.

## Introduction

The most effective method for the treatment of paroxysmal atrial fibrillation (AF) is pulmonary vein isolation (PVI).^1^ However, the likelihood of recurrence and the requirement for a redo procedure, with all of its possible implications, are still high.^2^ The effectiveness of radiofrequency (RF) point-by-point PVI significantly increased with the advent of contact force (CF) sensing ablation catheters.^3,4^ Recently, new markers for lesion prediction have been utilized, like the ablation index. The procedural success rate was further increased by routine application of these parameters.^5–7^ Nonetheless, AF recurrence, which is mostly brought on by PV reconnection, continued to be a challenge.^8^ As a result, more progress is necessary to increase PVI durability and enhance procedural safety by optimizing lesion prediction. Parameters such as CF, ablation time, and power are all factors that the ablation index takes into account when calculating a statistical probability of lesion quality. Nevertheless, local impedance (LI) drop, a significant biophysical marker of lesion formation, is not considered.^5,9,10^ A new CF sensing ablation catheter that can quantify LI changes has entered the market with pre-clinical data available.^11^

Recent research investigated the role of the LI drop in the formation of successful lesions during RF ablation for paroxysmal AF using the INTELLANAV STABLEPOINT catheter (Boston Scientific, Marlborough, MA, USA). It has been demonstrated that LI drop-guided ablation strategy is a highly accurate predictor of acute PVI segment conduction block in patients with paroxysmal AF. Furthermore, comparable LI drop cut-off values that predict acutely successful lesions have been identified,^12,13^ but still, no research has yet been done on the earliest signs of a successful lesion creation guided by this measurement.

Previous research has demonstrated that considerable oedema production can occur during ablation,^14^ and some recurrence has been linked to the development of this oedema and, consequently, reversible block.^15^ Due to reversible PV isolation, ablation-induced left atrial (LA) oedema may lead to procedural failure. This is because when the first ablation is partial, oedema develops, making the second ablation less effective^16^ hence highlighting the importance of a successful first application. In light of these findings, the present study intends to evaluate the LI trend as an indication of a successful lesion; find an optimal early cut-off value for the LI-guided ablation; and find an optimal time cut-off value for abandoning a lesion preventing unnecessary oedema creation.

## Methods

### Procedural workflow

Following the current guidelines,^1^ patients with symptomatic, drug-refractory paroxysmal AF were scheduled for PVI. Procedures were carried out with patients being sedated consciously. After femoral vein puncture and fluoroscopy-guided double transseptal puncture, a high-density mapping system (Rhythmia Mapping System, Boston Scientific, Marlborough, MA, USA) and a 64-electrode basket mapping catheter (INTELLAMAP ORION, Boston Scientific, Marlborough, MA, USA) were used to perform an anatomical map of the LA. As soon as the first transseptal puncture was made, intravenous unfractionated heparin was given to maintain an activated clotting time of greater than 300 s. By using a steerable sheath (Agilis, S or M curve; Abbott, Abbott Park, Illinois, USA) and a 4 mm tip, irrigated, CF sensing ablation catheter (StablePoint catheter, Boston Scientific, Marlborough, MA, USA), point-by-point RF applications were delivered around the antra of the ipsilateral PVs. To avoid touching the catheters during RF sessions and prevent data distortion (e.g. CF, LI), the basket catheter was positioned in the contralateral PVs during ablation. With a maximum temperature of 43°C, RF energy was applied in power control mode (40–50 W), characteristic of the high-power short-duration method. During ablation, the irrigation rate was 30 mL/min; during mapping, it was 2 mL/min. Automatic tagging (AutoTags) with 6 mm diameter tags was utilized. Numbered AutoTags were used to automatically mark the ablation points for tracking purposes. We applied overlapping ablation locations; hence, the inter-tag distances were less <6 mm between all nearby points. Predefined AutoTag settings were as follows: catheter stability (3 mm for >3 s), minimum CF (30% of time > 3 g), and minimal LI drop > 3 Ω. Based on the LI decrease, the AutoTag colouring was configured to be as follows: white 10 Ω, pink 10–20 Ω, and red >20 Ω. The LI decline was tracked throughout the applications to determine how long the ablation sessions should last. Based on results from prior experiments published,^11^ we sought to achieve a LI decrease of 20–30 Ω.

To validate acute lesion efficacy, bipolar pacing at the distal electrode pairs of the ablation catheter was performed along the entire ablation line, according to each ablation point, previously marked and numbered with AutoTags during ablation. Pacing was done with an output of 10 mA at 2 ms pulse duration with the intent to assess the success of the first-pass RF lesions, after the circumferential ablation line around the PVs antra had been completed, as previously described.^13^ The inability to capture the tissue at the designated ablation site was used to identify successful RF applications. Unsuccessful lesions were characterized as those that continued to be able to capture the surrounding tissue. Both successful and failing lesions’ AutoTag numbers were noted and the characteristics of the lesions were analysed accordingly. Further RF applications were delivered to unsuccessful sites until unexcitability was attained. The electrical conduction within the PVs was then examined after mapping the left and right ipsilateral PVs using the basket catheter. To complete the PVI if LA to PV conduction was present, RF applications that targeted conduction gaps on the circumferential line were applied. Entrance and exit block were confirmed 20 min after the last RF application by analysing the signals picked up by the basket catheter within each PV and by performing pacing procedures with the ablation catheter. In order to verify that the isolation line was completed additional 3D high-density voltage map of the LA was made.

### Data collection

With the aid of a local electric field created at the catheter’s tip, the ablation catheter utilized in this study can measure LI in real-time. The mapping system records trace-level data at very high sampling rate such as the change in CF, LI value and catheter position coordinates in 3D, as well as power, minimum CF, maximum CF, mean CF, duration of application, baseline LI, duration of the application, and session LI drop. Acquiring this information from the mapping system needs a special level of permission. Therefore, Semmelweis University has signed a ‘Rhythmia Data Use agreement’ with the company in order to gain access to this level of data. The export consists of a database storing vast information about each RF application point.

The ablation operation, data extraction, and analysis were all consented in writing by all patients. The Semmelweis University Regional and Institutional Committee of Science and Research Ethics accepted the study protocol, which complied with the Declarations of Helsinki (no. 280/2020).

### Data pre-processing

Study database was created by excluding points that had an error during data readout as well as CF and LI value outliers. All remaining numbered AutoTag points were exported from the database using MATBAL scripts to a file containing the following information: timestamp, filtered LI values, filtered CF values, and 3D coordinates of the tip of the catheter. Important indicators to be used in statistical analysis have been calculated from the file, such as mean force, force range, baseline LI, and LI value at pre-specified timings during ablation (2, 4, 5, 6, 8, 10, and 12 s). We characterized the LI for each time point with the mean of the five impedance values closest in time. Subtracting the base LI with the current LI value enabled the calculation of LI drop at the required timings. Also, the sum of the range of each 3D coordinate in the *x*, *y*, and *z* positions was analysed showing the spatial resolution of the catheter position in order to evaluate catheter tip stability during energy delivery.

### Statistical analysis

As the majority of the variables showed non-parametric distributions after performing the Shapiro–Wilk test, the continuous variables were expressed as medians with interquartile ranges, and the categorical variables were expressed as percentages with event numbers. The continuous variables were compared with the Mann–Whitney test and the Kruskal–Wallis test with *post hoc* analysis, as appropriate. We used the receiver operating characteristic (ROC) analysis to determine the optimal cut-off values for insufficient LI drop predicting unsuccessful applications. We tested the success of applications by using univariate logistic regression analysis. The multivariate logistic regression model was built in a forward stepwise manner; the baseline model included all relevant parameters. In our study, a two-tailed *P* < 0.05 was considered statistically significant. Statistical analyses were performed using IBM SPSS 25 (Apache Software Foundation, USA) and GraphPad Prism 8.1 (GraphPad Softwares Inc., USA), software products.

## Results

### Lesion characteristics and study population

In this current analysis, 643 applications were analysed, in 8 patients, 559 were successful and 84 were unsuccessful. The detailed characteristics of the study population and the basic parameters of the lesions were described previously. No steam pop occurred during the RF applications.^13^

**Figure 1:**
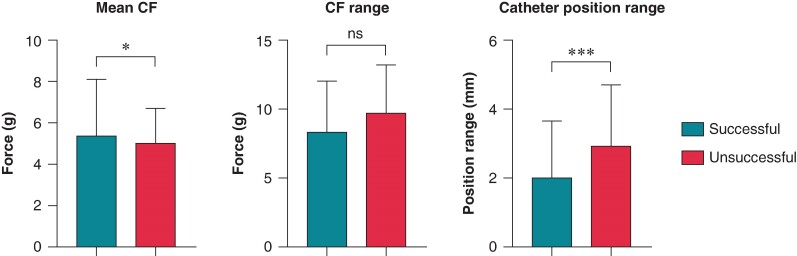
Comparison of catheter parameters in successful and unsuccessful applications. Mean CF, CF range, and catheter position range were compared using the Mann–Whitney test. Catheter position range: the sum of the range of exact catheter position in *x*, *y*, and *z* dimensions during the application; CF, contact force; *P < 0.05; ***P < 0.001; ns *P* > 0.05.

Due to the extended data export, much larger amount of raw data was available per ablation point; thus, the examined force and catheter position values were updated. There was a slight but statistically significant difference in mean CF (*P* = 0.04), while the CF range did not differ between groups (*P* = 0.245). The catheter position range was significantly higher in unsuccessful applications (*P* = 0.0006). The data are shown in *Table 1* and *Figure 1*.

**Table 1:** Description of the successful and unsuccessful lesions

Characteristic	Successful lesions	Unsuccessful lesions	*P*-value
Mean CF (g)	5.3 (3.4–8.1)	5.0 (3.1–6.7)	**0.041**
CF range (g)	8.3 (6.3–11.9)	9.7 (6.2–13.16)	0.245
Catheter position range (mm)	2.0 (1.1–3.6)	2.9 (1.6–4.7)	**0**.**0006**

Data is expressed as median with interquartile range. Catheter position range: the sum of the range of exact catheter position in *x*, *y*, and *z* dimensions during the application. CF, contact force. Bold values indicate statistically significant difference (*P* < 0.05).

#### Temporal description of local impedance drop during radiofrequency application

**Figure 2:**
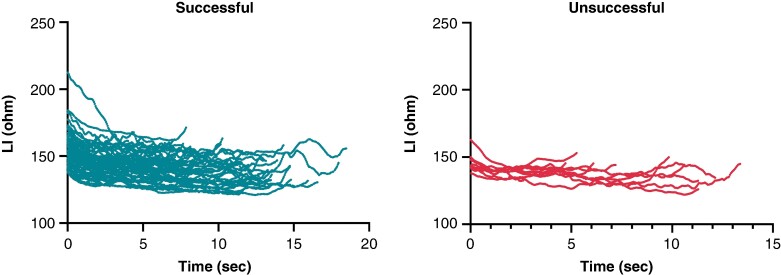
Sample data of local impedance of successful and unsuccessful applications from one randomly selected patient with 5 ms definition. LI, local impedance.

**Figure 3:**
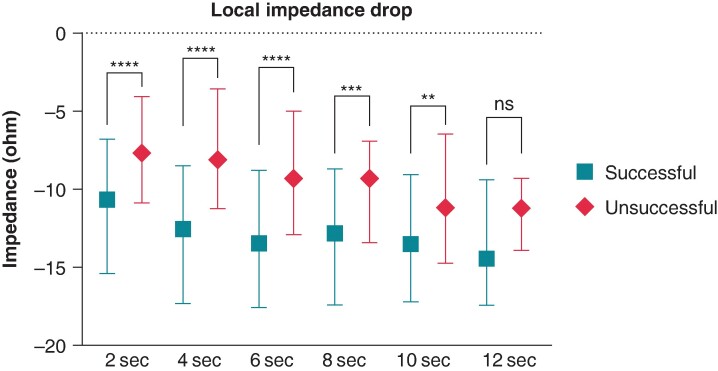
Local impedance drop of successful and unsuccessful applications. LI drop of each time point was compared using the Mann–Whitney test. **P < 0.01; ***P < 0.001; ****P < 0.0001; ns *P* > 0.05.

**Figure 4:**
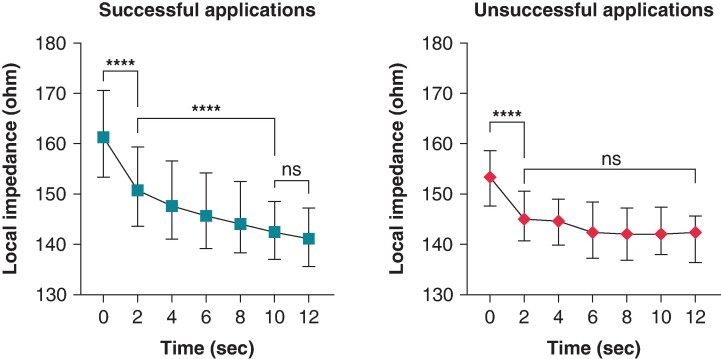
Change in LI during successful and unsuccessful applications. Local impedance was compared using the Kruskal–Wallis test. ***P < 0.001; ****P < 0.0001; ns *P* > 0.05.

Ultra–high-resolution analysis of the LI was also performed. Sample data of successful and unsuccessful applications from one patient with 5-ms definition is presented in *Figure 2*. Compared with the unsuccessful ablation points, the successful applications were characterized by a higher LI at baseline (*P* < 0.0001) and significantly higher LI drops at the 2-, 4-, 6-, 8-, and 10-s time points of RF ablation (*Table 2* and *Figure 3*). When analysing the temporal LI changes of unsuccessful applications, after a moderate but statistically significant drop from baseline to the 2-s time point (baseline 153 Ω vs. 2 s 145 Ω, *P* < 0.001), LI did not change further over time (2 s 145 Ω vs. 12 s 143 Ω; *P* = 0.99). On the other hand, successful applications’ LI after the significantly higher initial drop further decreased significantly up to the 10-s time point, respectively (baseline 161 Ω vs. 2 s 150 Ω vs. 10 s 141 Ω, *P* < 0.001 for all; *Figure 4*).

**Table 2:** Baseline local impedance and local impedance drop values of the successful and unsuccessful lesions

Local impedance (Ω)	Successful lesions	Unsuccessful lesions	*P*-value
Baseline	161.3 (153.4–170.6)	153.4 (147.7–158.6)	**<0.0001**
2-s drop	10.66 (6.78–15.39	7.63 (4.07–10.87)	**<0**.**0001**
4-s drop	12.54 (8.47–17.32)	8.10 (3.57–11.23)	**<0**.**0001**
6-s drop	13.47 (8.81–17.56)	9.30 (5.01–12.90)	**<0**.**0001**
8-s drop	12.81 (8.69–17.39)	9.27 (6.91–13.43)	**0**.**0006**
10-s drop	13.53 (9.06–17.21)	11.15 (6.42–14.74)	**0**.**0043**
12-s drop	14.45 (9.39–17.45)	11.20 (9.29–13.91)	**0**.**073**

Data is expressed as median with interquartile range. Bold values indicate statistically significant difference (*P* < 0.05).

#### Cut-off value for insufficient local impedance drop predicting unsuccessful applications

**Figure 5:**
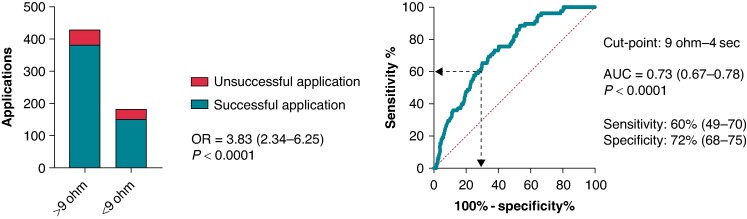
Impact of LI drop at the 4-s time point on application success. Receiver operating characteristic analysis was performed for determining the optimal cut-off point for insufficient LI drop predicting unsuccessful applications. The odds ratio refers to the presence vs. the absence of LI drop below 9 Ω at the 4 s time point. We tested the success of applications by using logistic regression analysis. HR, hazard ratio; OR, odds ratio; AUC, area under the curve.

To establish an optimal cut-point for the temporal pattern of LI drop during ablation that suggests unsuccessful applications, we used the ROC analysis. Local impedance drop below 9 Ω at the 4-s time point seemed to be an optimal cut-point indicating unsuccessful applications [AUC = 0.73 (0.67–0.78), *P* < 0.0001; sensitivity 60% (49–70); specificity 72% (68–75), positive predictive value 17% (13–22), negative predictive value 89% (87–91)]. Afterwards, we created groups of patients with LI drop below and over the previously established cut-points. We analysed the predictive value of the LI drop, the mean CF, the CF range, and the catheter position range on success of applications with logistic regression analysis. Failing to reach at least 9 Ω impedance drop at the 4-s time point predicted unsuccessful applications [OR 3.82 (2.34–6.25); *P* < 0.0001]. This is shown in *Figure 5*. The mean CF (*P* = 0.010) and the catheter position range (*P* = 0.006) proved to be significant predictors of success as well. At the next step, to verify the importance of the LI drop values, we analysed the parameters in a multivariate logistic regression model. Inadequate LI drop independently predicted unsuccessful applications after adjustment to mean CF, CF range, and catheter position range [OR 3.27 (1.84–5.82); *P* < 0.0001]. The detailed results of logistic regression analyses are presented in Supplementary material online, *Table S1*.

## Discussion

### Synopsis of key findings

Successful applications were characterized by significantly higher baseline LI and larger LI drop during the ablation. In case of unsuccessful applications, after a moderate but significant initial drop, LI did not change further over time. In contrast, in case of successful applications, we observed continuing, significant drop of the LI. Failing to reach at least 9 Ω impedance drop at the 4-s time point independently predicted unsuccessful applications, after adjustment to relevant factors.

#### Lesion creation with high-power ablation and validation of efficacy

High-power, short-duration ablation was recently introduced for performing PVI, resulting in favourable efficacy and safety characteristics with significantly decreased procedure times.^17^ The lesion geometry changes consequently due to the predominant role of resistive heating, causing shallower lesions, while transmurality is nonetheless achievable reliably in the LA.^18–20^ Acute lesion efficacy was validated with the loss of pace capture technique, which was previously shown to be effective in recognizing conduction gaps.^21^ Additionally, using it for verification of PVI improved the long-term success as well.^22^

#### The predictive role of local impedance drop in lesion formation

Measuring LI during RF catheter ablation proved to be a sensitive marker of adequate catheter–tissue coupling, the main determinant of energy delivery. In case of insufficient contact between the ablation catheter and the myocardium, RF current is transferred in the direction of the blood pool, hindering the heating of the myocardial tissue.^23,24^

Traditionally used parameters like generator impedance, CF, and surface temperature assess coupling with variable success and some limitations.^9,25^ Combining LI and CF measurement with this present novel ablation catheter *in vitro* and *in vivo* models provided information on tissue characteristics and catheter angulation as well, while improving RF energy titration. Importantly, LI drop during ablation predicted lesion depth and width with better correlation than force–time integral and generator impedance drop,^11^ mainly because it reflects the mechanism of real-time lesion formation more closely than any other parameter. The efficacy of high-power energy setting in the mid-term was also verified in a pre-clinical model.^26^

Our group provided the first clinical data with the INTELLANAV STABLEPOINT ablation catheter on the relevance of LI drop in predicting acute lesion formation.^13^ Successful applications were significantly shorter and had higher baseline LI and LI drops as well. Moreover, distinct cut-off values of LI drop were determined to predict the efficacy of applications on the anterior and posterior wall. The findings were parallel with the results of the LOCALIZE clinical trial, performed with the non-CF sensing IntellaNav MiFi OI ablation catheter,^27^ and another more recent paper dealing with LA box isolation.^12^

#### The temporal characteristics of local impedance drop during ablation and the correlation with acute lesion efficacy

In this study, we presented the first clinical data on the changes of LI during high-power RF ablation and characterized the differences between successful and unsuccessful applications. A continuous, significant drop was observed in case of successful applications all along the whole duration of RF ablation, which phenomenon was showed in the pre-clinical studies with this catheter as well.^11,26^ However, in case of unsuccessful applications, we detected a modest drop at the start of the RF application; after that, the LI did not change further during ablation. Interestingly, we only observed a minimal, clinically insignificant difference in CF and the CF range did not differ between the groups. Nevertheless, the baseline LI was significantly lower in unsuccessful applications that is often observed in scar tissue and is known to be a predictor of poor lesion formation. The range of the catheter position was greater during the unsuccessful ablation points, which can suggest worse catheter stability, also resulting in decreased LI according to previous studies.^11,26,28^ Both phenomenon may serve as the cause of insufficient RF ablation.

The clinical safety of high-power RF ablation is proven, the different lesion geometry also appears to be favourable to reduce thermal damage of surrounding tissues; however, the optimal titration of delivered RF energy is still warranted to prevent serious complications.^29–32^ Our previous results showed that monitoring LI during the application is useful to predict lesion efficacy and, therefore, able to prevent creating unnecessarily high LI drops and potentially risky lesions. A pre-clinical study with INTELLANAV STABLEPOINT ablation catheter highlighted that lesions that resulted in a steam pop were characterized with higher baseline impedance and larger LI drops. The probability of steam pop occurrence was 36% at LI drops of 65 Ω and a rapid increase was observed with even higher LI drops.^11^

Based upon the results of this study, inadequate early LI drop during the RF application might predict unsuccessful lesions, independently from CF and parameters of catheter stability. These applications probably should be timely interrupted to avoid needless energy delivery and ablation could be restarted after catheter repositioning, further improving the efficacy and safety of the procedure.

### Limitations

The main limitation of our study is the single-centre design, the relatively small sample size, although the number of analysed RF lesions, and LI data proved to be sufficient for conclusive statistical analysis. Since real-time LI measurement during RF ablation is available with the ablation system and catheter described above, the results cannot be extrapolated for other systems. In case of using LI values, it is not possible to determine a clear cut-off value that could verify the irreversible cardiomyocyte destruction, although adequate LI drop might predict successful lesions. No follow-up data were included in the investigation, and longer-term durability of RF applications could be validated with invasive remapping after the initial procedure. Therefore, the direct clinical impact should be strengthened with further clinical studies with higher number of included subjects.

## Conclusion

A rapid and enduring drop of the LI may predict effective lesion creation, while slightly changing or unchanged LI is associated with unsuccessful applications. A moderate LI drop during the first 4 s of RF application independently predicts ineffective RF delivery.

## Supplementary Material

euae260_Supplementary_Data

## Data Availability

The data underlying this article will be shared on reasonable request to the corresponding author.
