# Supplementary material for: Early rapid local impedance drop is associated with acute lesion efficacy during pulmonary vein isolation
Source: Europace. 2024 Oct 7;26(10):euae260. doi: 10.1093/europace/euae260 (PMC11493088; doi:10.1093/europace/euae260)
Supplement: euae260_Supplementary_Data [file euae260_supplementary_data.docx]

Supplementary Table S1. Multivariate logistic regression prediction model of unsuccessful applications

|  | Univariate logistic regression | | | | Multivariate logistic regression | | | |
| --- | --- | --- | --- | --- | --- | --- | --- | --- |
| Characteristics | OR | 95% CI | χ2 | p | OR | 95% CI | χ2 | p |
| Sum of position range | 1.21 | 1.07-1.37 | 9.37 | 0.002 |  |  |  |  |
| Mean force (g) | 0.92 | 0.86-0.99 | 5.74 | 0.017 |  |  |  |  |
| Force range (g) | 1.01 | 0.97-1.03 | 0.01 | 0.900 |  |  |  |  |
| Inadequate LI drop | 3.83 | 2.34-6.25 | 28.67 | <0.0001 | 3.27 | 1.84-5.82 | 16.17 | <0.0001 |

OR: odds ratio; CI: confidence interval; χ^2^: chi squared.

The multivariate model included sum of position range, mean force and force range.
